# Detection of early lung cancer among military personnel (DECAMP) consortium: study protocols

**DOI:** 10.1186/s12890-019-0825-7

**Published:** 2019-03-07

**Authors:** Ehab Billatos, Fenghai Duan, Elizabeth Moses, Helga Marques, Irene Mahon, Lindsey Dymond, Charles Apgar, Denise Aberle, George Washko, Avrum Spira, on behalf of the DECAMP investigators

**Affiliations:** 1 Division of Pulmonary, Allergy, and Critical Care Medicine, Boston University School of Medicine, Boston, MA 02118 USA; 2 Department of Biostatistics, Brown University, Providence, RI 02912 USA; 3 Division of Computational Biomedicine, Boston University School of Medicine, Boston, MA 02118 USA; 4 American College of Radiology Imaging Network, Philadelphia, PA 19103 USA; 5 Department of Radiological Sciences, University of California at Los Angeles, Los Angeles, CA 90024 USA; 6 Division of Pulmonary and Critical Care Medicine, Brigham and Women’s University, Boston, MA 02115 USA

**Keywords:** Lung cancer, Biomarker, Gene expression, DECAMP

## Abstract

**Background:**

Lung cancer is the leading cause of cancer-related death due in large part to our inability to diagnose it at an early and potentially curable stage. Screening for lung cancer via low dose computed tomographic (LDCT) imaging has been demonstrated to improve mortality but also results in a high rate of false positive tests. The identification and application of non-invasive molecular biomarkers that improve the performance of CT imaging for the detection of lung cancer in high risk individuals would aid in clinical decision-making, eliminate the need for unnecessary LDCT follow-up, and further refine the screening criteria for an already large high-risk population.

**Methods:**

The Detection of Early Lung Cancer Among Military Personnel (DECAMP) consortium is conducting two multicenter prospective studies with the goals of developing an integrated panel of both airway and blood-based molecular biomarkers that discriminate benign and malignant indeterminate nodules detected on CT scan as well as predict the future development of lung cancer in high-risk individuals. To achieve these goals, DECAMP is compiling an extensive array of biospecimens including nasal brushings, serum, plasma and intrathoracic airway samples (bronchial brushings and bronchial biopsies) from normal-appearing airway epithelium.

**Discussion:**

This bank of samples is the foundation for multiple DECAMP efforts focused on the identification of those at greatest risk of developing lung cancer as well as the discrimination of benign and malignant pulmonary nodules. The clinical, imaging and biospecimen repositories will serve as a resource for the biomedical community and their investigation of the molecular basis of chronic respiratory disease.

**Trial registration:**

Retrospectively registered as NCT01785342 - DECAMP-1: Diagnosis and Surveillance of Indeterminate Pulmonary Nodules (DECAMP-1). Date of Registration: February 7, 2013.

Retrospectively registered as NCT02504697 - DECAMP-2: Screening of Patients With Early Stage Lung Cancer or at High Risk for Developing Lung Cancer (DECAMP-2). Date of Registration: July 22, 2015.

## Background

Lung cancer is currently the leading cause of cancer-related death. In 2017, there were an estimated 222,500 new cases of lung cancer and 155,870 deaths from lung cancer in the U.S. alone [1]. Despite significant advances in treatment for lung cancer patients, the 5-year survival rate in the United States remains at 15.6% [2]. The low survival rate is due, in large part, to our inability to diagnose lung cancer at an early stage - more than half of lung cancer cases are diagnosed at the time when an individual has advanced disease [1].

While the National Lung Screening Trial (NLST) demonstrated a 20% relative reduction in mortality from lung cancer using Low-Dose Computed Tomography (LDCT), most of the 1.5 million indeterminate pulmonary nodules found annually on chest computed tomography (CT) are benign [3, 4]. As a result, there are safety concerns about potential complications from diagnostic procedures, the financial and infrastructural burden of diagnostic follow-up from false-positive screens, and the undue anxiety and cancer-treatment associated morbidity for asymptomatic indolent tumors [5, 6]. Furthermore, less than 27% of individuals diagnosed with lung cancer in the US would have met NLST criteria to be eligible for screening [5]. Additional biomarkers are needed to improve our ability to detect and intervene on lung cancer at its earliest and potentially most curable stage.

Significant advances have been made evaluating molecular alterations in the pathogenesis of a wide spectrum of malignancies including breast, prostate and colon cancer [7–9]. In lung cancer, there are efforts to develop biomarkers for disease detection using proteomics, epigenetics, and both DNA and RNA from the airway, as well as molecular signals in other tissue compartments [10–26]. Clinical application of non-invasive molecular biomarkers for lung cancer could aid in clinical decision-making, eliminate the need for unnecessary LDCT follow-up, and further refine the screening criteria for an already large high-risk population.

One challenge in translating early detection biomarkers into the clinic is the lack of prospective screening and diagnostic cohorts for biomarker discovery and validation in their intended use populations [27–30]. This is compounded by lack of available resources and the need for multiple biospecimens from different body compartments that can be used as singular features or as an integrated panel in concert with imaging. The Detection of Early Lung Cancer Among Military Personnel (DECAMP) consortium is conducting multicenter prospective studies to determine the diagnostic accuracy of molecular and imaging-based biomarkers in the airway and blood for detecting lung cancer in the presence of an indeterminate nodule (DECAMP-1) as well as develop an integrated model (i.e. clinical, imaging, and molecular markers) that results in a robust diagnostic predictor for lung cancer. The consortium is also focused on the discovery of molecular biomarkers in the airway and blood for preclinical detection of lung cancer using a longitudinal screening cohort (DECAMP-2). These studies represent unique multicenter cohorts of high-risk smokers that can be leveraged for the investigation of lung cancer and other chronic respiratory diseases by the research community.

## Methods

### Study design

**Fig. 1:**
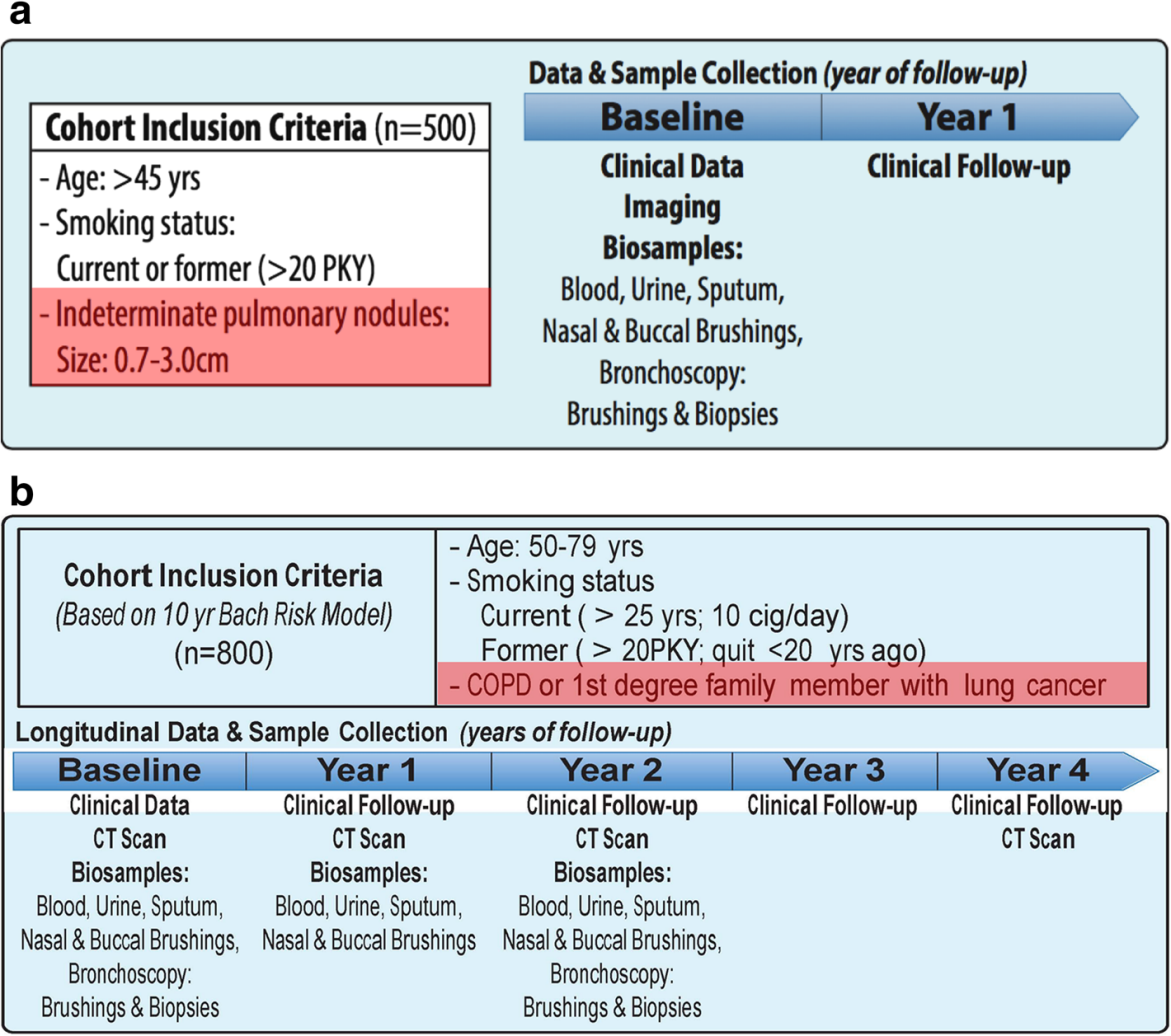
**a** DECAMP-1 Schema. DECAMP-1 is recruiting 500 subjects aged 45 and older with indeterminate pulmonary nodules (0.7 to 3.0 cm) and a 20+ pack-year smoking history. Patients in this cohort have biospecimens collected at baseline and then are followed prospectively until a diagnosis of cancer or benign is made. This is the diagnostic arm of the study. **b** DECAMP-2 Schema. DECAMP-2 is recruiting 800 subjects aged 50–79 with a 20+ pack-year smoking history and a diagnosis of chronic obstructive pulmonary disease (COPD), emphysema or family history of lung cancer. Biospecimens from these patients are collected at baseline and annually. This constitutes the screening arm of the study

DECAMP is a multi-center consortium comprised of 15 military treatment facilities, Veterans Affairs (VA) hospitals, and academic centers across the United States (Tables 1 and 2). It has undertaken two separate clinical protocols, DECAMP-1 (NCT01785342) and DECAMP-2 (NCT02504697). DECAMP-1 is recruiting 500 subjects aged 45 and older with indeterminate pulmonary nodules (0.7 to 3.0 cm) and a 20+ pack-year smoking history (Fig. 1a). DECAMP-2 is recruiting 800 subjects aged 50–79 with a 20+ pack-year smoking history and a diagnosis of chronic obstructive pulmonary disease (COPD), emphysema or family history of lung cancer (Fig. 1b). These subjects are being identified through primary care and dedicated lung nodule clinics as well as specialty clinics in pulmonary, thoracic surgery and thoracic oncology.

**Table 1:** Participating institutions

Military hospitals	VA hospitals	Academic hospitals
Walter Reed National Military Center	Boston VA Healthcare System	Boston University
Naval Medical Center Portsmouth	North Texas VA Medical Center	UCLA Health
Naval Medical Center San Diego	Denver VA Medical Center	University of Pennsylvania
Brooke Army Medical Center	Los Angeles VA Healthcare System	Roswell Park Cancer Institute
	Tennessee VA Healthcare System	
	Philadelphia VA Medical Center	
	Pittsburgh VA Healthcare System

**Table 2:** Inclusion/Exclusion criteria

DECAMP-1	
Inclusion Criteria	
• 45 years of age or older	
• Radiologic diagnosis of indeterminate pulmonary nodule (0.7 to 3.0 cm); must be of appropriate size at enrollment, but nodule(s) may have been first identified within 12 months prior; if multiple nodules were diagnosed, choose the one with the longest diameter as the target lesion; if two or more nodules are of the same largest size, choose the one with the perpendicular longest diameter	
• CT scan completed within 3 months prior to enrollment	
• Smoking status: Current or former cigarette smoker with ≥20 pack years (pack years = number of packs per day X number of years smoked)	
• Willing to undergo fiberoptic bronchoscopy	
• Able to tolerate all biospecimen collection as required by protocol	
• Able to comply with standard of care follow up visits including clinical exams, diagnostic work-ups, and imaging for approximately 2 years from enrollment	
• Able to fill out Patient Lung History questionnaire	
• Willing and able to provide a written informed consent	
Exclusion Criteria	
• History or previous diagnosis of lung cancer	
• Diagnosis of pure ground glass opacities for the target lesion on chest CT (i.e., mixed features on the target lesion and pure ground glass opacity on non-target lesions are acceptable)	
• Contraindications to nasal brushing or fiberoptic bronchoscopy, including: ulcerative nasal disease, hemodynamic instability, severe obstructive airway disease, unstable cardiac or pulmonary disease, inability to protect airway, or altered level of consciousness	
• Allergies to any local anesthetic that may be used to obtain biosamples in the study	
DECAMP-2	
Inclusion Criteria	
• Ages 50 to 79 years	
• Smoking status: Current or former cigarette smoker (≥10 cigarettes/day for at least 25 years’ duration for current smokers, or ≥ 20 pack years for former smokers who quit 20 years ago or less)	
• History of Chronic Obstructive Pulmonary Disease (COPD), emphysema, or at least one first-degree relative with a diagnosis of lung cancer	
• Willing to undergo fiberoptic bronchoscopy	
• Able to tolerate all biospecimen collection as required by protocol	
• Able to comply with standard-of-care follow-up visits, including clinical exams, diagnostic work-ups, and imaging for a maximum of 4 years or until diagnosis of lung cancer	
• Able to fill out Patient Lung History questionnaire	
• Willing and able to provide a written informed consent	
Exclusion Criteria	
• Diagnosis of lung cancer prior to the current assessment	
• Contraindications to nasal brushing or fiberoptic bronchoscopy, including: ulcerative nasal disease, hemodynamic instability, severe obstructive airway disease (i.e., disease severity does not allow for bronchoscopic procedures), unstable cardiac or pulmonary disease, as well as other comorbidities leading to inability to protect airway, or altered level of consciousness	
• Pre-existing pulmonary nodule(s) only if the treating physician determines the nodule presents a risk for cancer	
• Allergies to any local anesthetic that may be used to obtain biosamples in the study	
• Weight greater than that allowable by the CT scanner

### Inclusion criteria – DECAMP-1

- 45 years of age or older
- Smoking status: Current or former cigarette smoker with ≥20 pack-year exposure
- Radiologic diagnosis of indeterminate pulmonary nodule (0.7 to 3.0 cm); must be of appropriate size at enrollment, but nodule(s) may have been first identified within 12 months prior; if multiple nodules were diagnosed, choose the one with the longest diameter as the target lesion; if two or more nodules are of the same largest size, choose the one with the perpendicular longest diameter
- CT scan completed within 3 months prior to enrollment

### Exclusion criteria – DECAMP-1

- Previous diagnosis of lung cancer
- Diagnosis of pure ground glass nodules for the target lesion on chest CT (note: a target lesion of part-solid consistency in addition to a non-target pure ground glass nodule is acceptable)

### Inclusion criteria – DECAMP-2

- Ages 50 to 79 years
- Smoking status: Current or former cigarette smoker (≥10 cigarettes/day for at least 25 years’ duration for current smokers, or ≥ 20 pack years for former smokers who quit 20 years ago or less)
- History of COPD, emphysema, or at least one first-degree relative with a diagnosis of lung cancer

### Exclusion criteria – DECAMP-2

- Diagnosis of lung cancer prior to the current assessment
- Pre-existing pulmonary nodule(s) only if the treating physician determines the nodule presents a risk for cancer

### Study organization

**Fig. 2:**
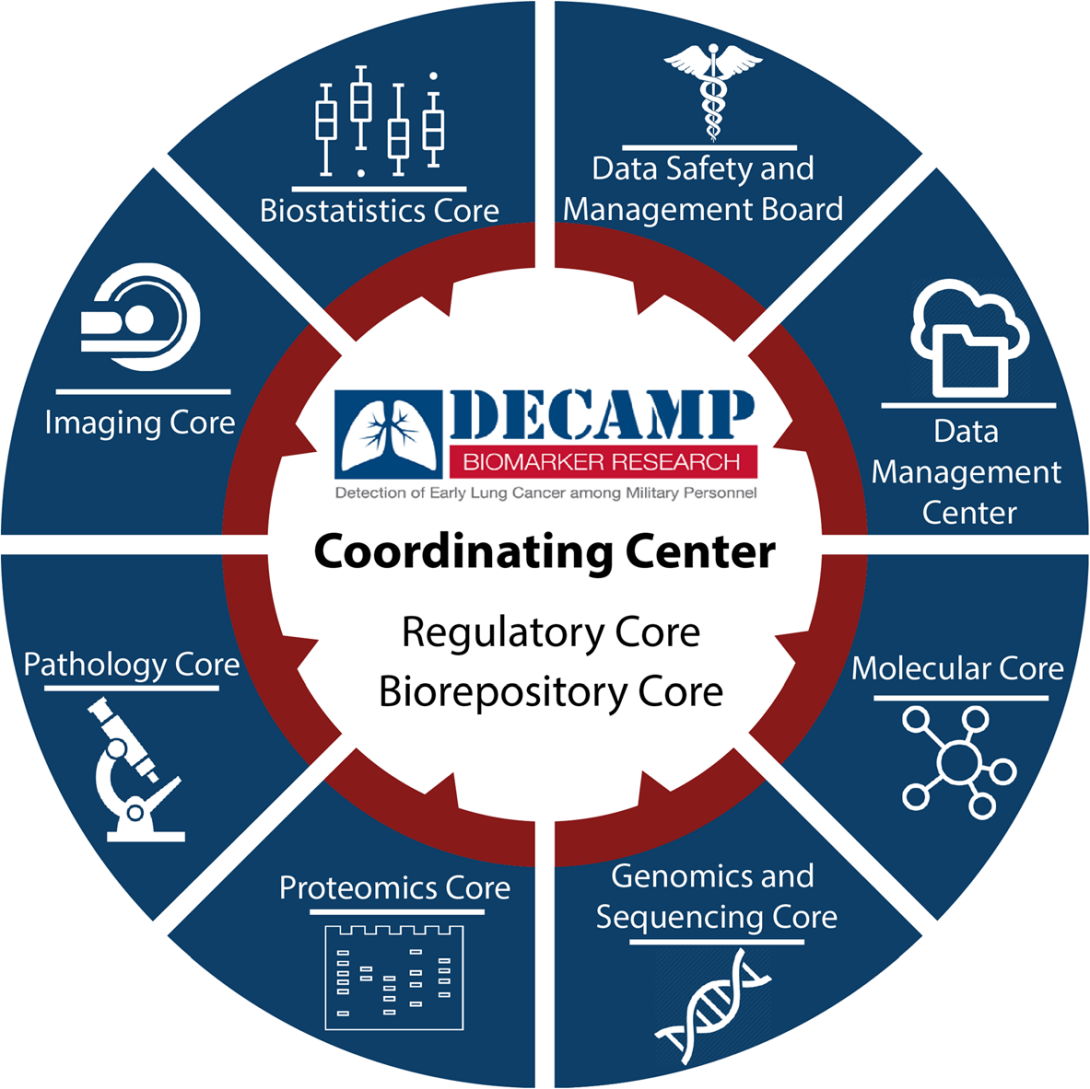
DECAMP Study Group Infrastructure. The DECAMP consortium is a multidisciplinary and translational research program that includes 15 clinical study sites (7 VA hospitals, 4 designated Military Treatment Facilities [MTF], and 4 academic hospitals), several molecular biomarker laboratories, and Biostatics, Bioinformatics, Pathology, and Biorepository core centers and laboratories. The DECAMP Coordinating Center serves as the primary administrative facility which regulates the design and execution of the study within this multi-institutional consortium

The DECAMP consortium is a multidisciplinary and translational research program that includes 15 clinical study sites (7 VA hospitals, 4 designated Military Treatment Facilities [MTF], and 4 academic hospitals), several molecular biomarker laboratories, and Biostatics, Bioinformatics, Pathology, and Biorepository core centers and laboratories (Fig. 2). The DECAMP Coordinating Center facilitates rapid selection, design, and execution of clinical studies within this multi-institutional consortium. The American College of Radiology Imaging Network (ACRIN) Biostatistics and Data Management Center (BDMC) is responsible for data collection and management, data monitoring and reporting, and data analysis. The DECAMP Clinical Consortium Steering Committee (CCSC) regularly reviews the overall study accrual and may request information about a site’s accrual performance to better understand general accrual barriers or issues. Accrual, safety information, and adverse events are presented to the DECAMP Data and Safety Monitoring Board (DSMB) at regularly scheduled meetings. Finally, the Biorepository Core is responsible for receiving and tracking biospecimens as well as performing quality assurance and quality control measures on archived specimens.

### Clinic visit

Subjects who meet inclusion and exclusion criteria are approached for informed consent to participate in the study in accordance with IRB regulations. Upon completed consent, they are enrolled and asked to complete a Lung Health Questionnaire. The Lung Health Questionnaire covers basic demographics, personal medical history, family medical history, medications, smoking history, alcohol and recreational drug history, and symptom history such as cough, dyspnea, and sputum production. All CT imaging and pulmonary function test data is collected and subjects subsequently undergo biospecimen acquisition including nasal brushings, buccal scrapings, sputum, blood and urine. A bronchoscopy is then performed to obtain endobronchial brushings and endobronchial biopsies. In DECAMP-1, these subjects are subsequently followed per routine standard of care for their indeterminate pulmonary nodule until they achieve a final diagnosis of cancer or no cancer (up to 2 years). In DECAMP-2, participants are followed until the last subject enrolled has been observed for 4 years. Bronchoscopic specimens are obtained in the 1st and 3rd years of this period of observation while the remainder of the biospecimens are obtained in the 1st, 2nd, and 3rd years. Clinical data is collected yearly for the full time of enrollment. Those individuals diagnosed with lung cancer are followed for an additional 3 years to monitor treatment strategies and progression. The DECAMP protocols do not specify routine standard of care, which is at the discretion of the site and/or provider and patient.

### Biospecimen acquisition

**Fig. 3:**
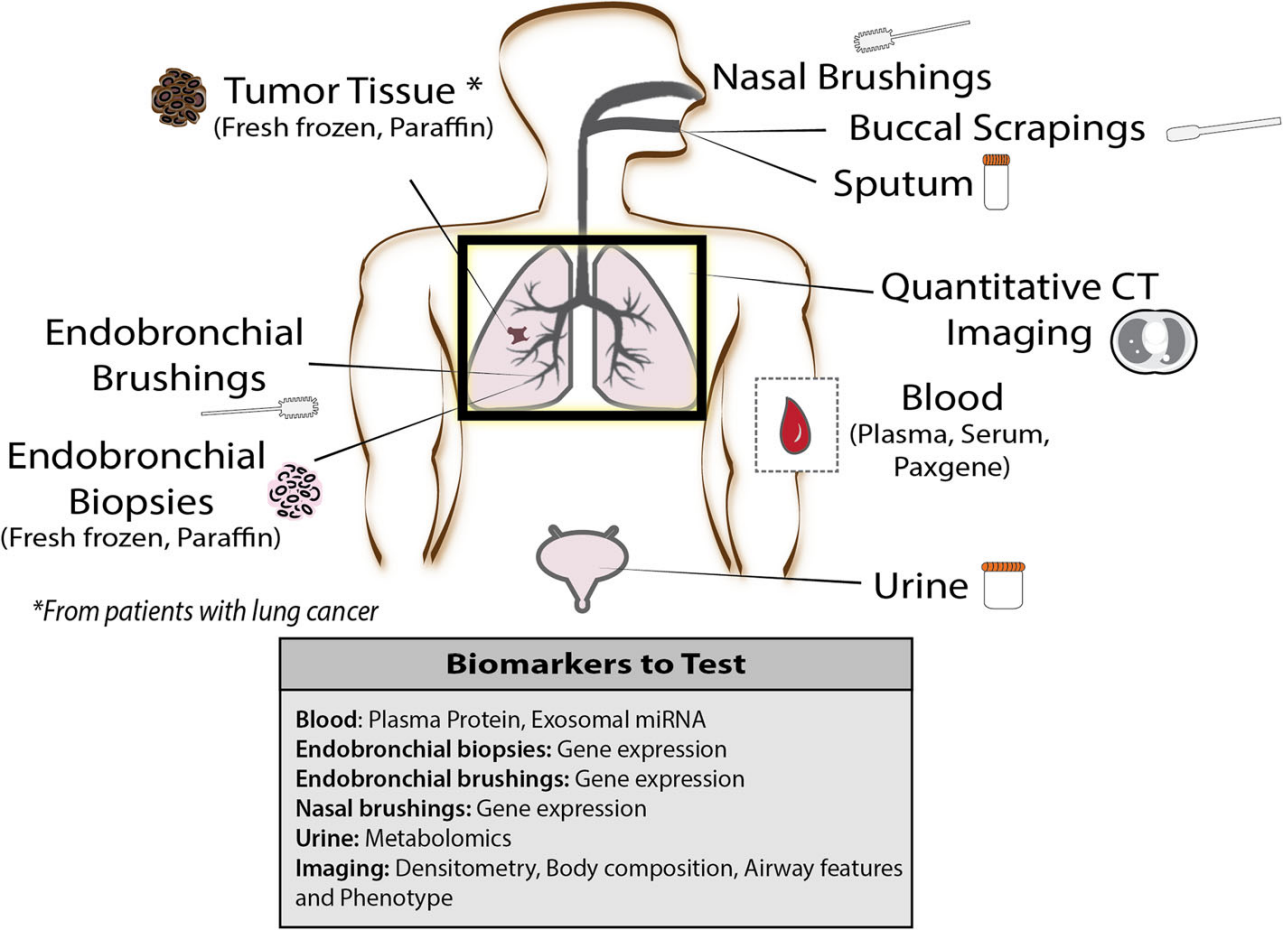
Biomarker Map. Bronchoscopy is performed at baseline in both DECAMP-1 and DECAMP-2 and a second time after 2 years for participants enrolled in DECAMP-2 to capture bronchial brushings and endobronchial forceps biopsies. Other specimens collected include nasal brushings, buccal scrapings, blood (fractionated into plasma and serum), urine, and sputum samples. Computed Tomography (CT) imaging is collected on all patients and used for quantitative CT imaging techniques. In patients who have a confirmed diagnosis of lung cancer and qualify as surgical candidates, fresh frozen and formalin-fixed paraffin-embedded tumor tissue is collected as well

Methods for biospecimen collection are detailed in the DECAMP ACRIN Biospecimen Procedure Manual. Bronchoscopy is performed at baseline in both DECAMP-1 and DECAMP-2 and a second time after 2 years for participants enrolled in DECAMP-2. Three bronchial brushings are obtained from the right mainstem bronchus and six endobronchial forceps biopsies are collected from the right upper lobe (two), right middle lobe (two), and left upper lobe (two). Other specimens collected at baseline include serum, urine, and sputum samples. Two nasal brushings are collected with the assistance of a nasal speculum from the left nostril and five buccal scrapings are collected from alternating sides of the mouth. A summary of biospecimens collected is shown in Fig. 3.

### Imaging acquisition

DECAMP-1 utilizes CT scans that are collected as part of routine clinical care while DECAMP-2 utilizes a standardized protocol for image acquisition and reconstruction. These volumetric scans are collected using low radiation dose helical techniques on a minimum 16-slice multidetector scanner. The same display field of view is utilized for each individual participant’s follow-up visits and scans. The scans are acquired at 2.5 to 5 mm but are reconstructed into 1.25 mm slice thickness using standard and high spatial frequency convolution kernels. Images from all sites are de-identified and submitted to the ACR Imaging Core Laboratory for storage.

### Molecular biomarkers

Given the unique set of biospecimens being collected within DECAMP, there is the opportunity to both discover novel molecular and imaging biomarkers in the screening and diagnostic setting as well as validate existing biomarkers. The endobronchial brushes in DECAMP-1 will enable validation in the indeterminate nodule setting of a previously developed gene-expression classifier that has been shown to improve the diagnostic performance of bronchoscopy among intermediate risk patients [31]. The nasal brushings will be leveraged to develop and refine a nasal gene-expression biomarker for both the diagnostic and screening setting [32]. A recently discovered urinary metabolomic biomarker [33] will be refined and validated in DECAMP-1. Serum and plasma collection will be used to discover circulating miRNA, circulating tumor DNA and proteins that hold potential for detecting lung cancer. DECAMP will also provide the opportunity for integration of multiple biomarkers across multiple biospecimens, to achieve high sensitivity and specificity.

### Imaging biomarker

Qualitative and quantitative CT (QCT) analysis of the DECAMP scans includes the extraction of features in the lung parenchyma, airways and chest wall. Semantic features using a common ontology with illustrated examples will be used to assess the relative performance of visual versus quantitative feature classification of indeterminate lung nodules. Densitometric assessments of emphysema in the parenchyma are performed to provide measures of the percentage of low attenuating tissue less than select Hounsfield Units (i.e. %LAA-950, %LAA-910) as well as the Hounsfield Unit (HU) threshold that defines the lowest attenuating 10th and 15th percentiles of lung (PERC10, PERC15, [34, 35]). Additional parenchymal subtyping will be performed using a local histogram approach to feature classification to provide percentages of the lung with emphysema (both centrilobular and paraseptal), interstitial change (defined as reticular, ground glass, and nodular), and visually normal appearing tissue [36–38]. Objective assessments of central airway morphology will be performed in the apical segment of the right upper lobe and the posterobasal segment of the right lower lobe [39]. Airway features extracted from the images will include the lumen area (LA), wall area (WA), wall thickness (WT) and the wall area percent (WA%). Finally, objective assessments of body composition will be extracted from the chest wall. These will include measures of the pectoralis muscle area (PMA) and subcutaneous fat adjacent to these muscles [40, 41]. Quantitative features of indeterminate nodules are typically based on segmentation of the nodule (the region of interest) and the use of various first and higher order statistics of the nodule pixel attenuation threshold; run-length features, which provide a measure of contiguous gray levels along a specific orientation; co-occurrence matrix features that describe the frequency of one gray-level intensity with another in a specified spatial relationship in a given range; Laws texture features; and wavelet features that decompose the 3D images into orthogonal components [42–45].

### Adjudication process to determine lung cancer diagnosis

A DECAMP Adjudication Committee will confirm lung cancer diagnosis in study participants. The role of the Adjudication Committee (AC) is to adjudicate and review diagnoses of pulmonary nodules as lung cancer and non-lung cancer cases in a consistent and unbiased manner throughout the course of the trial and is comprised of two pulmonologists with extensive experience in lung cancer diagnosis and adjudication processes.

### Statistical considerations

The goal of DECAMP-1 is to determine the diagnostic accuracy of genomic and proteomic biomarkers in the airway and blood to discriminate benign and malignant indeterminate pulmonary nodules identified on CT scan. The binary outcome of cancer/not cancer was selected for analysis of classification performance based on area under the receiver operating characteristic curve (AUC) analysis. At its study inception, the rate of cancer was conservatively assumed to be approximately 15% to assure that recruitment would ultimately achieve an adequate sample size to support multiple biomarker analyses. Interim analyses of the DECAMP-1 data suggests that 15% significantly underestimates the true rate at which indeterminate nodules will be found to be malignant thus ensuring that the study is adequately powered for multiple biomarker analyses.

The goal of DECAMP-2 is to discover new molecular markers in the airway and blood that predict future lung cancer. We hypothesized that there will be approximately 50 markers with at least a two-fold difference between participants who develop lung cancer over the period of observation and those that do not. Using methods outlined by Jung and colleagues we determined that at least 50 subjects in DECAMP-2 would need to develop lung cancer to provide 85% power at a false discovery rate less than 5% [46, 47].

## Discussion

DECAMP is a consortium of multi-disciplinary investigators recruiting cohorts of study subjects to develop and validate molecular and imaging-based biomarkers for early lung cancer detection. DECAMP-1 will identify biomarkers that can be used to discriminate the etiology of indeterminate pulmonary nodules and DECAMP-2 will identify smokers at greatest risk for future lung cancer. The major strength of this consortium lies within its extensive efforts focused on molecular characterization including biospecimens collected from the blood, upper respiratory tract and intrathoracic airway samples (bronchial brushings and bronchial biopsies). These data will allow detailed investigation of peripheral and organ specific biologic pathways that may ultimately be used for cancer detection and prognostication in the clinical setting.

DECAMP can also be leveraged to investigate other conditions such as lung disease secondary to chronic tobacco smoke exposure. It is increasingly clear that the presence of lung diseases like COPD or pulmonary fibrosis are associated with an increased risk of lung cancer in smokers but the molecular basis for the shared susceptibility of these observations is not completely understood. The clinical and radiologic data being collected in DECAMP will support multiple investigations of lung disease including those defined by the presence of expiratory airflow obstruction, restriction, or CT based features such as interstitial and emphysematous remodeling of the lung parenchyma. In addition to the planned biospecimen analyses focused on lung cancer, additional banked specimens can also be utilized to establish the association between lung specific and peripheral omics based investigation. Such work would augment ongoing National Heart, Lung, and Blood Institute (NHLBI) supported efforts in cohorts such as COPDGene and SPIROMICS.

There are a number of important limitations to DECAMP. The cohort is enriched for male military personnel where the prevalence and burden of smoking is much higher than in the general population [48–50]. This results in a study cohort that is at approximately twice the risk of developing lung cancer as the general population [48, 49]. While such enrichment will augment the planned biomarker analyses, it may also limit the translation of DECAMP based biomarkers to the general population of smokers. Furthermore, DECAMP-1 is biased to patients with an indeterminate nodule who are undergoing a clinically indicated bronchoscopy. This will both enrich the cohort for subjects who have an increased pretest risk of having cancer and will limit the study cohort to those that are deemed to be clinically stable enough to undergo bronchoscopy. Lastly, DECAMP is a longitudinal observational study in which the sickest participants are at greatest risk for drop out and loss to follow up.

The DECAMP investigators are actively recruiting for two investigations that will provide new data to help determine the risk of lung cancer, impact subsequent screening protocols in at risk patients and potentially impact therapeutic decision making as the field continues to adopt efforts focused on precision medicine. The aggregate of these efforts will address an unmet clinical need and help transition cancer care to curing disease at its earliest stages and ultimately preventing lung cancer in at risk smokers.

## Abbreviations

AC: Adjudication Committee
ACRIN: American College of Radiology Imaging Network
AUC: Area under the receiver operating characteristic curve
BDMC: Biostatistics and Data Management Center
CCSC: Clinical Consortium Steering Committee
COPD: Chronic obstructive pulmonary disease
CT: Computed tomography
DECAMP: Detection of Early Lung Cancer Among Military Personnel
DSMB: Data and Safety Monitoring Board
HU: Hounsfield Unit
LA: Lumen area
LDCT: Low dose computed tomographic
MTF: Military treatment facilities
NHLBI: National Heart, Lung, and Blood Institute
NLST: National Lung Screening Trial
PMA: Pectoralis muscle area
QCT: Quantitative computed tomography
VA: Veterans Affairs
WA: Wall area
WA%: Wall area percent
WT: Wall thickness
